# Pathological Technique

**Published:** 1898-01

**Authors:** 


PATHOLOGICAL TECHNIQUE. A practical manual for the pathological laboratory. By FRANK B. MALLORY, A. M., M. D., Assistant Professor of Pathology, Harvard University Medical School; Assistant Pathologist to the Boston City Hospital; Pathologist to the Children's Hospital and the Carney Hospital, Boston, and James H. Wright, A. M., M. D, Director of the Laboratory of the Massachusetts General Hospital; Instructor in Pathology, Harvard University Medical School. Octavo, 397 pages, with 105 illustrations. Philadelphia: W. B. Saunders, 925 Walnut street. 1897.

This book is designed especially for practical use in pathological laboratories, both as a guide to beginners and as a source of reference for the advanced. The book will also meet the wants of practitioners who have opportunity to do general pathological work.

The work is divided into three parts—Part I. being devoted to post mortem examinations, Part II. to bacteriological examinations and Part III. to histological methods, both for normal and pathological histology. The chapters on post mortem examinations are carefully and fully written, even going into the minutiæ of details. As a rule too little time is given in medical curricula to this most important subject, and the physician's knowledge is generally derived from personal experience. The authors insist upon thoroughness in all details of the autopsy and the importance they give to this subject is praiseworthy and timely. Very often the life and liberty of an individual depends upon the findings of an autopsy, and when inexperienced operators perform the work misinterpretation of the conditions present may do the accused party great injustice.

The authors have not given all the different methods and formulæ employed in the laboratories, but have chosen those which have been found of the greatest service in practical work.

The illustrations are well chosen, nicely executed and carefully described. The index is very complete, adding greatly to the value and accessibility of the different methods. The book is well printed on good, heavy paper, and is a welcome addition to libraries of laboratory workers. W. C. K.

